# Supplementary material for: Vegetation Affecting Water Quality in Small Streams: Case Study in Hemiboreal Forests, Latvia
Source: Plants (Basel). 2022 May 16;11(10):1316. doi: 10.3390/plants11101316 (PMC9142884; doi:10.3390/plants11101316)
Supplement: Supplementary file 1 [file plants-11-01316-s001.zip › plants-1728214-supplementary.pdf]

Supplementary material S1

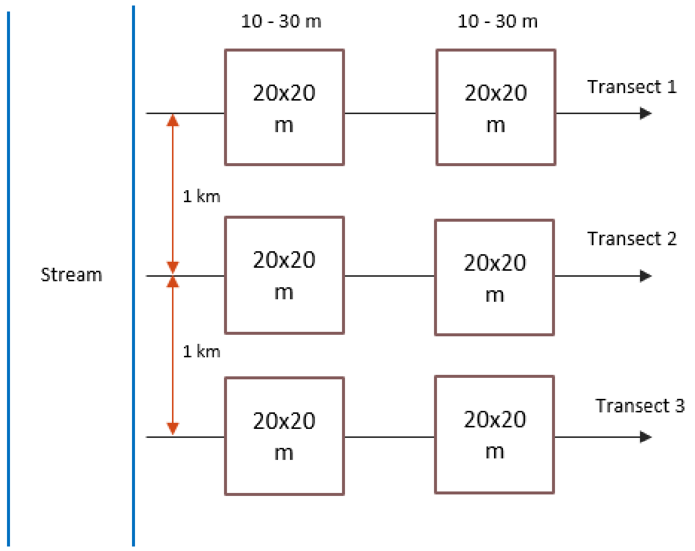

Figure S1. Scheme of sample plots. At each transect, we placed two sample plots with size of 20×20 m (400 m2) at a distance of 10 and 60 m from the water edge.

Supplement material S2

Table S1. Bioindicator analysis. Component A shows the probability that species are found in the sample plots that belong to the target site group (if component A = 1 then species are found only in the plots of the specific group). Conversely, component B shows the probability that species are found in all selected sites (if component B = 1 then species are found in all selected sample plots). _1- represent species from the shrub layer; _2 –represent species from the tree layer.

	A	B	stat	p value
Group High water quality				
Anemone nemorosa L.	0.74	0.51	0.62	0.008
Ulmus glabra Huds._2	0.87	0.39	0.58	0.001
Ulmus glabra Huds._1	0.70	0.39	0.52	0.042
Dicranum majus	0.99	0.24	0.52	0.007
Deschampsia caespitosa L.	0.97	0.22	0.52	0.036
Betula pubescens Ehrh.	1.00	0.17	0.52	0.010
Deschampsia flexuosa L.	1.00	0.15	0.52	0.018
Group Low water quality				
Alnus incana L.	0.61	0.50	0.55	0.018
Stellaria media L.	0.96	0.27	0.51	0.003
Galeopsis bifida Boenn.	1.00	0.23	0.48	0.003

Glechoma hederacea L.	0.87	0.23	0.45	0.011
Dactylis glomerata L.	0.83	0.17	0.37	0.034
Calamagrostis arundinacea L.	1.00	0.10	0.32	0.041
Group Medium water quality				
Viola mirabilis L.	0.94	0.44	0.65	0.001
Rubus caesius L.	0.91	0.39	0.60	0.004
Ranunculus lanuginosus L.	1.00	0.22	0.47	0.001
Ribes spicatum E.Robson	1.00	0.22	0.47	0.002
Chelidonium majus L.	1.00	0.17	0.41	0.008
Platgredzene platygyrium	0.88	0.17	0.38	0.012
Campanula trachelium L.	0.57	0.22	0.36	0.048
Salix alba L._1	1.00	0.11	0.33	0.045
Salix alba L._2	1.00	0.11	0.33	0.045
Geranium sylvaticum L.	1.00	0.11	0.33	0.045
Ribes uva-crispa L.	1.00	0.11	0.33	0.045
Group High - Medium water quality				
Picea abies L._1	0.86	0.79	0.83	0.01
Hylocomium splendens	0.94	0.44	0.64	0.03
Viburnum opulus	0.98	0.25	0.50	0.029
Group High - Low water quality				
Mercurialis perennis L.	1.00	0.28	0.53	0.038
Group Medium - low water quality				
Ribes alpinum L.	0.85	0.65	0.74	0.003
Aegopodium podagraria L.	0.99	0.52	0.72	0.001
Urtica dioica L.	0.87	0.48	0.65	0.002
Euonymus europaea L.	0.80	0.46	0.61	0.014
Angelica sylvestris L.	0.84	0.42	0.59	0.019
Equisetum sylvaticum L.	0.94	0.35	0.58	0.002
Carex sylvatica Huds.	0.80	0.38	0.55	0.032
Humulus lupulus L.	0.99	0.27	0.52	0.003
Festuca arundinacea Schreb.	0.92	0.25	0.48	0.024
